# Recovery of Oligomeric Proanthocyanidins and Other Phenolic Compounds with Established Bioactivity from Grape Seed By-Products

**DOI:** 10.3390/molecules24040677

**Published:** 2019-02-14

**Authors:** Federica Pasini, Fabio Chinnici, Maria Fiorenza Caboni, Vito Verardo

**Affiliations:** 1 Interdepartmental Centre for Agri-Food Industrial Research, University of Bologna, via Quinto Bucci 336, 47521 Cesena (FC), Italy; federica.pasini5@unibo.it (F.P.); fabio.chinnici@unibo.it (F.C.); maria.caboni@unibo.it (M.F.C.); 2 Department of Agricultural and Food Sciences, University of Bologna, Piazza Goidanich 60, 47521 Cesena (FC), Italy; 3 Department of Nutrition and Food Science, University of Granada, Campus of Cartuja, 18071 Granada, Spain; 4 Institute of Nutrition and Food Technology (INYTA) ‘José Mataix’, Biomedical Research Centre, University of Granada, Avenida del Conocimiento s/n, E-18071 Granada, Spain

**Keywords:** grape seeds, flavan-3-ols, oligomeric proanthocyanidins, phenolic compounds, LH-20 Sephadex

## Abstract

Grape seeds are a copious part of the grape pomace produced by wine and juice industry and they represent an interesting source of phenolic compounds. Proanthocyanidins (PAs) are the main class of grape seed phenols and are important dietary supplements for their well-known beneficial properties. In this study enriched extracts obtained from Chardonnay and Pignoletto grape seeds were characterized for their proanthocyanidins and other minor phenolic compounds content and composition. Seed PAs were fractionated using Sephadex LH-20, using different ethanol aqueous solutions as mobile phase and analysed by normal phase HPLC-FLD-ESI-MS. Monomers, oligomers up to dodecamers and polymers were recorded in all samples. For both cultivars, the extracts showed a high content in PAs. The determination of other phenolic compounds was carried out using a HPLC-QqQ-ESI-MS and Chardonnay samples reported a greater content compared to Pignoletto samples. Contrary to PAs fraction, extracts obtained with ethanol/water 50/50 (*v*/*v*) presented a significant higher phenolic content than the others.

## 1. Introduction

Grape crops are one of the main extended agro economic activities in the world. Grape-derived products industry, such as winery and juices industries, generates high amounts of pomaces that include pulp residues, stems, skins and seeds. It has been estimated that the grape pomace amounts are approximately 20% (*w*/*w*) of wine or juice industry [1]. Grape pomace represents an environmental problem; however, it contains large amounts of phytochemicals (phenolic compounds among others), because of that it could be considered a low-cost source of these bioactive compounds [2].

Grape seeds consist of 13% of the grape’s weight and represent from 38% to 52% (dry weight) of grape pomace [3,4]. Its composition is very interesting due to the content of 40% dietary fibre, 16% oil, 11% protein and 7% phenolic compounds and other substances.

After pressing, grape seeds are still a rich source of oil and bioactive compounds, like polyphenols. The major part of grape seeds phenols are proanthocyanidins, a class of compounds which take form of oligomers and polymers derived from flavan-3-ols units, linked mainly by 4- 8 or 4-6 bonds [5]. Previous studies report the highest concentration of these compounds in grape seeds compared to grape skins and steams [5,6]. Furthermore, grape seeds present only the procyanidin-type of proanthocyanidins, consisting of (+)-catechin and (−)-epicatechin units. Monomers are the most abundant flavan-3-ols in these seeds, even if procyanidin oligomers and polymers, until high degree of polymerization (DP), were also detected. Another characteristic of the flavanol composition of grape seeds is the presence of derivatives esterified with gallic acid [5,7,8].

The growing interest in by-products as source of bioactive compounds has prompted the attention of the investigators on grape seed procyanidins and their beneficial effects on human health [9]. Several studies report the free radical scavenging and antioxidant activity of grape seed proanthocyanidins, demonstrating higher bioavailable and greater protection than vitamins C, E and β-carotene [10,11]. Besides the antioxidant potential, grape seed proanthocyanidins exhibit cardioprotective effects, preventing atherosclerosis as antioxidants of human low density lipoprotein [12]. Research studies show also anticarcinogenic [13,14,15,16], anti-inflammatory [17] and antiulcer [18] activity of the grape seed phenolic extract, as well as preventing the progression of cataract formation [19].

Sephadex LH-20 resin is frequently employed for proanthocyanidin purification and fractionation [20,21]. Acetone was usually used as elution solvent in order to recover the proanthocyanidins from Sephadex LH-20 resin; however, several studies [22,23,24] showed that ethanol could be a good alternative in order to obtain flavan-3-ols enriched extracts. Particularly, Li et al. [23] reported that 55/45 ethanol/water (*v*/*v*) solution is able to recover gallocatechin-gallate; at the same time, Tian et al. [24] showed that 70/30 and 90/10 ethanol/water (*v*/*v*) eluted fractions contain flavan-3-ol compounds like catechin and procyanidins.

On the basis of these previous studies, the aim of this work was to compare the purification efficiency of the ethanol/water solution (50/50 and 80/20 (*v*/*v*)) in order to obtain proanthocyanidin enriched extracts from two grape seed by-products. Moreover, other goal was to characterize the proanthocyanidin profile and other minor phenolic compounds in enriched extracts purified by resins. The oligomeric and polymeric flavan-3-ols fractions were characterized by normal-phase HPLC-FLD-MS, whereas the other phenolic compounds were analysed by reverse-phase HPLC-QqQ-MS.

## 2. Results

### 2.1. Separation and Identification of Oligomeric Proantocyanidins

As previously described, literature on the use of ethanol solutions to recover flavan-3-ols from LH-20 Sephadex resin is scarce. However, Li et al. [23] using a Sephadex LH-20 gel and 55% ethanol–water solution as eluent allowed an extract of gallocatechin-gallate (GCG) with a 91% of purity and a recovery of 68% from *Camellia ptilophylla*. Nevertheless, Tian et al. [24] studied the recovery of flavan-3-ols from different Finnish berry plants after purification with Sepahadex LH-20 using 0, 20, 40, 70 and 90% ethanol as eluent solutions; their results underlined that the highest recovery of flavan-3-ols was obtained eluting with 70–90% of ethanol. Thus, taking into account these previous results, two solutions (50 and 80 % of aqueous ethanol) were used as eluent to recover flavan-3-ols from grape seed by-products.

**Figure 1:**
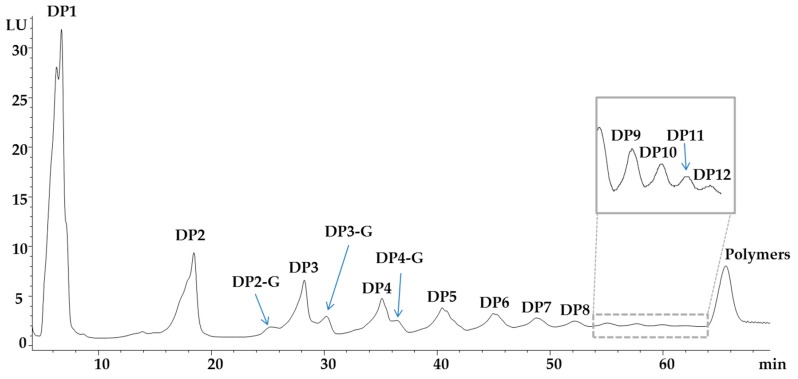
Grape seed extracts flavan-3-ols profile as obtained by normal phase HPLC separation.

The dried extracts obtained after resin purification were analysed by HPLC-FLD-ESI-MS in order to determine the oligomeric flavan-3-ols. Figure 1 shows the HPLC chromatogram of the flavan-3-ols and their analytical parameters are reported in Table 1.

Peak at 6.7 min resulted in [M-H]^−^ ion at *m*/*z* 289 that is attributed to catechin and epicatechin that were described in grape seeds by several authors [25,26]. Their presence has been confirmed by co-elution with chemical standards. Peak at 18.2 min reported a [M-H]^−^ ion at *m*/*z* 577 that has been assigned to procyanidin dimers; Prodanov et al. [25] described the presence of several dimer isomers such as PC B1, PC B2, PC B3, PC B4, PC B5 and PC B6 in Malvar grape seeds. An ion peak was detected at *m*/*z* 729 (retention time 25 min) which value has been previously attributed to the mass of a galloylated procyanidin dimer [25,26]. [M-H]^−^ ion at m/z 865 was detected for the peak at 28.1 min; therefore this ion peak was attributed to procyanidin trimer according to literature [25,26]. Peak at 30.4 min showed two majors [M-H]^−^ ion at *m*/*z* 881 and 1017 corresponding to galloylated procyanidin trimers. Peak eluting at 35.3 min, showing [M-H]^−^ at *m*/*z* 1153 was identified as procyanidin tetramer according to Prodanov et al. [25]. Compound eluting at 36.8 min showed [M-H]^−^ at *m*/*z* 1305 and was identified as monogalloylated procyanidin tetramer. Three co-eluting compounds at 41.2 min with [M-H]^–^ 1441, 797 and 873 *m*/*z* were identified respectively, no-galloylated and galloylated procyanidin pentamers. Procyanidin oligomers from 6 to 12 degrees of polymerization were assigned comparing the grape seed extract chromatogram with a co-elution of an apple sample. Finally, the peak at 65.7 min was attributed to polymers flavan-3-ols (> 12 of degree of polymerization) [26].

### 2.2. Quantification of Oligomeric Proantocyanidins

The concentrations of monomers and proanthocyanidins (PAs) identified in the different grape seed extracts are reported in Table 2. The normal phase HPLC analysis with fluorimetric detection and diol stationary phase permitted the separation and quantification of the proanthocyanidins in distinct peaks, according to their degree of polymerization (DP). As shown in Table 1, in all the fractions obtained from Chardonnay (C) and Pignoletto (P) grape seeds, monomers, oligomers up to dodecamers and polymers were recorded. In agreement with a previous study [26], monomers represented the principal flavan-3-ols present in the grape seed samples, accounting for more than 60% of the total PAs content. For both cultivars, the extracts obtained eluting ethanol/water 80/20 *v*/*v* (CF1 ad PF1) showed a significant higher content than the fraction eluted with ethanol/water 50/50 *v*/*v* (CF2 and PF2). These results confirmed that high alcohol level released less soluble and more stable compounds such as flavan-3-ols [27]. The same trend was observed for dimers content, with CF1 as the richest sample. Dimers amount was about the 10%, whereas trimers and tetramers were less abundant with an amount from 3.6 to 4.1% and from 2.3 to 2.8%, respectively. As already reported elsewhere [26], with increasing DP the concentration of oligomers decreased until less than 1% from octamers to dodecamers. Polymers varied in a range from 3.3 to 6.6% of the total PAs, showing a similar concentration in all extracts, except for PF1. Finally, the total flavan-3-ols (SPAs: sum of monomers, oligomers and polymers) followed the trend of monomers and dimers, with CF1 as the most concentrated sample and CF2 the less one. These results strongly agree with the data reported by Tian et al. [24] that showed as higher ratio of ethanol were able to recover high amounts of (+)-catechin, (−)-epicatechin and B-type procyanidin dimers in sea buckthorn berry and crowberry and several leaf extracts (sea buckthorn, saskatoon, white currant, lingonberry, hawthorn). The same authors also noticed that ethanol/water 60/40 (*v*/*v*) allowed lower recovery of flavan-3-ols only in Saskatoon and hawthorn leaf extracts, confirming the low extraction power of the solvent when high amounts of water is present in the elution solvent.

Galloylated dimers, trimers and tetramers were also found and they eluted after their non-galloylated PA, with a significant lower amount. Their total content (SGPAs) was similar in the grape seed extracts (about 24 mg/g), except for CF2 (18.7 mg/g); nevertheless, their percentage content on the total PAs amount was up to 6% for extracts CF2, PF1 and PF2, whereas CF1 showed a percentage of about half of the others (3.8%). Their presence in grapes seeds is usually evaluated after hydrolysis and expressed as percentages of galloylated units [28]. With this analytical approach, the % of galloylation in grape seeds has been found to spans between 13% and 30% depending on the PAs polymerization degree [28,29]. The lower percentages found in our samples is certainly due to: (i) the different analytical technique we adopted, capable of estimating the absolute amount of procyanidins gallate instead of single gallic residues; (ii) the lack of chromatographic separation of polymeric PAs > 12 DP.

Although the trend was not the same for all individual compounds, CF1 and PF1 showed higher total content in PAs compared to the others. Polymers were up 6% of total proanthocyanidins in F2 fractions and less than 4% in F1 fractions. It is, however, worth to note that, while for Pignoletto seeds the use of the two extraction solvents (80/20 and 50/50) gave quite similar results in terms of composition of the extract, for Chardonnay samples, the solution composed of ethanol/water 80/20 *v*/*v* resulted in higher extraction of monomeric and oligomeric compounds, with likely distinct antioxidant and organoleptic characteristics with respect to other.

Grape seed proanthocyanidins garnered the attention of several researchers and companies due to their bioactive properties. Several investigations have been developed in the last years, focusing the attention as on technological use of procyanidins from winery by-products [30,31] than for functional foods and/or nutraceuticals [32,33,34,35,36,37,38].

As reported by several authors [39,40,41,42,43,44,45,46] one of the activities of grape proanthocyanidins is related to the amelioration of the symptoms of metabolic syndrome diseases and its related diseases. Different mechanisms were reported; Banerji and Banerjee [39] proposed that the control of type 2 diabetes mellitus in advanced-stage patients is possible using a mixture of grape seed procyanidin extract (GSPE); Indian gooseberry, turmeric and fenugreek extracts that is able to prevent β cell apoptosis and facilitate cell replication due to a decrease of the pancreatic oxidative stress and modulation of the immune response. Grape seed proanthocyanidin extract was also used by Sun et al. [42] in diabetic rats and they reported the reduction of apoptosis of retinal cells suggesting the protection of the retina against hyperglycaemic damage, probably due to the amelioration of oxidative stress-mediated injury via the activation of the Nrf2 pathway. Aragones et al. [41] demonstrated that grape seed proantocyanidins modulate many metabolic pathways in the liver in a dose-dependent manner increasing the NAD+ availability and activating SIRT1, which was significantly associated with improved protection against hepatic triglyceride accumulation. Moreover, as reported by Seo et al. [40], grape flavonoids reduced the hepatic ROS and prevents non-alcoholic fatty liver disease by reducing oxidative stress and inflammation; modulating cholesterol, bile acid and ceramide synthesis and lipid metabolism in the liver; and ameliorating insulin resistance. The body weight loss was also noticed; this effect was confirmed in vivo by Serrano et al. [47] that showed as GSPE improved the lipid oxidation in subcutaneous adipose tissue and consequently improved the total energy expenditure. Moreover, the inhibition of adipogenesis induced by grape procyanidin B2 and GSPE were also reported by others [45,48].

### 2.3. Identification and Quantification of Other Phenolic Compounds

While proanthocyanidins represent the largest part of grape seed flavonoid composition, other phenolic compounds, which have been reported in minor amounts, were also investigated for their contribution to the bioactive properties of grape seed extracts [49]. Accordingly, to determine other phenolic compounds present in the grape seed extracts, HPLC-QqQ-MS analysis has been carried out. Several phenolic compounds were identified and the analytical parameters for their analysis are reported in Table 3.

A total of five phenolic acid derivatives and five flavonols have been quantified using MRM mode. Briefly, MS/MS data permitted the identification of gallic acid with *m*/*z* 169 and fragment at 125 *m*/*z*; it was previously described in grape seeds and their extracts by several authors [25]. Protocatechuic aldehyde was also identified and its presence in grape seeds was reported by Prodanov et al. [25]. Two compounds at 463 *m*/*z* and fragment at 301 *m*/*z* were detected and according to Prodanov et al. [25] they were attributed to ellagic acid hexoside isomers; moreover, aglycone form of ellagic acid was detected [25]. Among the different flavonols, the peak with *m*/*z* 449 and fragment ions at 287 and 259 *m*/*z*, according to Prodanov et al. [25], was identified as dihydrofisetin glucoside. Kaempferol-glucoside with a pseudomolecular ion at *m*/*z* 447 and fragment at *m*/*z* 285 (kaempferol) was also detected [50]. On the other hand, the flavonols quercetin-3-pentoside (two isomers) and quercetin were detected; the pseudomolecular ion at 433 *m*/*z* and the fragments at 301, 179 and 151 *m*/*z* confirmed the presence of quercetin-pentoside [51]. Finally, quercetin was detected with pseudomolecular ion at *m*/*z* 301 and fragments at 179 and 151 *m*/*z* [50].

The quantification of the phenolic acid derivatives and flavonols present in the grape seed extracts is reported in Table 4, as µg/g of dry weight.

The ellagic acid hexoside isomers were the predominant phenolic compounds in all samples, accounting for more than 70% for the isomer 1 and only from the 12 to 17% for the isomer 2. Both of them were present at the highest amount in sample CF2, whereas PF1 had the lowest content compared to the other extracts. Gallic acid was the third most abundant compounds with amounts varying from 4.1 to 6.1%, with CF1 as the most concentrated sample. According to the results obtained by Garcia-Jares et al. [49] on 11 distinct monovarietal grape seed extracts, Chardonnay seed demonstrated to be particularly rich in this trihydroxylated and highly antioxidant phenolic acid. Ellagic acid showed also an important percentage in sample CF1 and CF2, whereas it was detected in quantities under the Limit of Detection (LOQ) for the extracts obtained from the Pignoletto seeds. All the other phenolic compounds accounted for less than 1%. The total content has the same trend observed for the main phenol (ellagic acid hexoside 1), where CF2 showed the highest quantity in the amount of 36,853.3 µg/g, followed in decreasing amount by CF1 (29,202.5 µg/g), PF2 (25,729.6 µg/g) and PF1 (16,056.8 µg/g).

These data show how the Chardonnay seed extracts reported a greater phenolic compound content compared to Pignoletto samples. In addition, contrary to proanthocyanidin results, the extracts obtained eluting ethanol/water 50/50 *v*/*v* (CF2 and PF2) presented a significantly higher phenolic content than the fraction eluted with ethanol/water 80/20 *v*/*v* (CF1 ad PF1). These data strongly agree with the results reported by other authors [9,27] that underlined as high water ratio allowed high phenolic acids recovery.

## 3. Materials and Methods

### 3.1. Extraction and Purification of Phenolic Compounds

Grape seeds were obtained from berries of Chardonnay (C) and Pignoletto (P) after wine production (Faenza, Italy, 44°17′00″N 11°53′00″E) and they were air-dried at room temperature (final moisture 13%) and grounded to a granulometry of 2 mm. Briefly, according to Ky and Teissedre [27] 25 g of seeds were extracted with 500 mL of water-ethanol (3/7 *v*/*v*) under sonication for 40 min in an ultrasound bath (Starsonic 90 Liarre (Bologna, Italy) equipment with frequency 34 kHz) and the solvent was rotary evaporated under vacuum at 35 °C to remove ethanol. The resulting extracts were washed two times with 250 mL of n-hexane to remove lipid-soluble substances and then rotary evaporated to remove the residual hexane. After that, the extract was fractionated using Sephadex LH-20. The aqueous fraction was applied to a Sephadex LH-20 column (20 × 450 mm) (GE Healthcare, Barrington, IL, USA) and the resin was previously equilibrated with water (150 mL). The extracts were obtained using two water-ethanol ratio solutions: extracts F1 were obtained eluting with 300 mL of ethanol/water 80/20 *v*/*v*, whereas extracts F2 were eluted with 300 mL of ethanol/water 50/50 *v*/*v*. Both fractions were immediately frozen at −20 °C and then freeze-dried (Thermo HETO, power dry LYOLAB 3000; Waltham, MA, USA) and stored at −23 °C until the analysis.

### 3.2. HPLC-FLD-ESI-MS Analyses of Oligomeric Proanthocyanidins

The final extracts were dissolved in water-ethanol (1/1, *v*/*v*), filtered through 0.45 μm PTFE syringe filters and analysed by HPLC (Agilent 1200 Series, Agilent Technologies, Palo Alto, CA, USA), equipped with a binary pump delivery system, a degasser, an autosampler and a fluorimetric detector (FLD) and coupled to a single quadrupole mass spectrometer (MSD, model G1946A, Santa Clara, CA, USA).

Proanthocyanidins were separated in a Develosil Diol 100Å column 5 m, 250 × 4.6 mm ID (Phenomenex, Torrance, CA, USA), according to Robbins et al. [52]. Fluorescence detection was conducted with an excitation wavelength of 230 nm and an emission wavelength of 321 nm. The injection volume was 5 µL and all the analyses were carried out at 35 °C. Calibration curves of (+)-catechin and procyanidin B2 were both arranged in the range of limit of quantification (LOQ)-500 and LOQ-500 µg/mL, respectively, at 6 concentration levels for each compound. The correction factors suggested by Robbins et al. [52] were used to quantify the oligomeric proanthocyanidins from trimers to dodecamers and polymers. The limit of detection (LOD) and the limit of quantification (LOQ) were 0.058 and 0.193 µg/mL, respectively, for catechin and 0.042 and 0.14 µg/mL, respectively, for procyanidin B2. LOQ was calculated based on the standard deviation (σB) of γ-intercepts of linear regression and the slope of the calibration curve (S) of standards, according to the formula: LOQ = 10 (σB)/S.

### 3.3. HPLC-QqQ-ESI-MS Analyses of Other Phenolic Compounds

The HPLC method established by Gomez-Caravaca et al. [53] was used. MRM analyses were performed on 6420 Triple Quadrupole (Agilent Technologies, Santa Clara, CA, USA) equipped with the Agilent HPLC 1200 series autosampler and a binary pump. Phenolic separation was performed on a 100 mm × 3.0 mm Zorbax Poroshell C18 column (Agilent Technologies, Millford, MA, USA) at 25 °C. MS/MS acquisition parameters (MRM mode) used for identification of the target phenolic compounds are provided in Table 2. The phenolic compounds were quantified as ellagic acid equivalents (ellagic acid and ellagic acid hexoside), rutin equivalent (quercetin pentoside and dehydrofisetin glucoside), quercetin equivalent and gallic acid equivalent (gallic acid and protocatechuic aldehyde). The calibration curves were built, from LOQ-500 mg/L, at six concentration levels, plotting peak area versus analyte concentration. LOQ was calculated based on the formula described in the Section 3.2.

### 3.4. Statistical Analysis

HPLC analyses were replicated three times for each extract and calibration point (*n* = 3). Significant differences (at *p* < 0.05) were explored by using analysis of variance (ANOVA) combined with the Tukey’s post-hoc test using Statistica 8.0 software (2007, StatSoft, Tulsa, OK, USA).

## 4. Conclusions

The results demonstrated that ethanol, a food grade solvent, is a good choice to recover the flavan-3-ol compounds from grape seed by-products. The elution with ethanol/water 80/20 (*v*/*v*) recovered the highest amounts of proanthocyanidins and Chardonnay seed extracts reported a greater flavan-3-ols content compared to Pignoletto samples. In addition, contrary to proanthocyanidin results, the extracts obtained eluting ethanol/water 50/50 (*v*/*v*) presented a significant higher phenolic content than the fraction eluted with ethanol/water 80/20 (*v*/*v*). Once again, these results suggest the possibility to modulate the quali-quantitative characteristics of bioactive compounds extracted from grape seeds, as a function of the composition of the eluent utilized.

## Figures and Tables

**Table 1:** Analytical parameters of HPLC–FLD-ESI-MS method.

Compounds	Retention Time (min)	[M-H]^−^	In Source Fragment (*m*/*z*)
Monomers (DP1)	6.7	289	245
Dimers (DP2)	18.2	577	425, 289
Galloylated dimers (DP2-G)	25	729	303
Trimers (DP3)	28.1	865	739, 713
Galloylated trimers (DP3-G)	30.4	881, 1017	593, 303
Tetramers (DP4)	35.3	1153 c	865
Galloylated tetramers (DP4-G)	36.8	1305	-
Pentamers (DP5)	41.2	1441, 797, 873	-
Hexamers (DP6)	45.2	-	-
Heptamers (DP7)	49	-	-
Octamers (DP8)	52.2	-	-
Nonamers (DP9)	55.0	-	-
Decamers (DP10)	57.8	-	-
Undecamers (DP11)	60.1	-	-
Dodecamers (DP12)	62.0	-	-
Polymers	65.7	-	-

**Table 2:** Concentrations (mg/g) of flavan-3-ols in grape seeds enriched extracts from cv. Chardonnay (C) and Pignoletto (P) berries. *: SGPAs = Sum of galloylated proanthocyanidins; SPAs = Sum proanthocyanidins.

Compounds	CF1	CF2	PF1	PF2
	PAs (mg/g)			
Monomers (DP1)	456.1 ± 3.4 ^a^	185.2 ± 2.9 ^d^	252.6 ± 3.0 ^b^	222.5 ± 8.3 ^c^
Dimers (DP2)	55.4 ± 0.1 ^a^	29.1 ± 0.02 ^d^	42.9 ± 0.2 ^b^	38.4 ± 0.01 ^c^
Galloylated dimers (DP2-G)	9.8 ± 0.1 ^a^	8.3 ± 0.3 ^b^	9.6 ± 0.1 ^a^	10.3 ± 0.1 ^a^
Trimers (DP3)	22.8 ± 0.01 ^a^	11.4 ± 0.1 ^c^	15.2 ± 0.1 ^b^	14.7 ± 1.5 ^b^
Galloylated trimers (DP3-G)	8.4 ± 0.01 ^a^	6.3 ± 0.02 ^b^	8.7 ± 0.3 ^a^	8.8 ± 0.0 ^a^
Tetramers (DP4)	14.6 ± 0.1 ^a^	8.3 ± 0.1 ^c^	9.9 ± 0.1 ^b^	9.2 ± 0.5 ^b,c^
Galloylated tetramers (DP4-G)	5.6 ± 0.1 ^a^	4.1 ± 0.0 ^c^	5.2 ± 0.1 ^b^	5.2 ± 0.2 ^b^
Pentamers (DP5)	11.7 ± 0.1 ^a^	6.4 ± 0.1 ^c^	8.0 ± 0.1 ^b^	7.9 ± 0.3 ^b^
Hexamers (DP6)	6.4 ± 0.1 ^a^	3.9 ± 0.0 ^d^	4.3 ± 0.02 ^c^	4.5 ± 0.01 ^b^
Heptamers (DP7)	6.5 ± 0.1 ^a^	4.5 ± 0.0 ^c^	4.6 ± 0.0 ^c^	4.9 ± 0.0 ^b^
Octamers (DP8)	4.2 ± 0.2 ^a^	3.2 ± 0.0 ^b^	3.2 ± 0.1 ^b^	3.4 ± 0.1 ^b^
Nonamers (DP9)	2.3 ± 0.02 ^a^	2.0 ± 0.02 ^b,c^	2.0 ± 0.02 ^c^	2.1 ± 0.03 ^b^
Decamers (DP10)	3.2 ± 0.01 ^a^	3.0 ± 0.02 ^b,c^	2.9 ± 0.02 ^c^	3.1 ± 0.02 ^a,b^
Undecamers (DP11)	3.0 ± 0.0 ^a^	2.9 ± 0.0 ^b^	2.8 ± 0.0 ^c^	3.0 ± 0.0 ^a,b^
Dodecamers (DP12)	2.9 ± 0.01 ^a^	2.8 ± 0.01 ^b^	2.7 ± 0.01 ^c^	2.9 ± 0.02 ^a^
Polymers	21.0 ± 0.2 ^a,b^	19.9 ± 0.7 ^b^	15.8 ± 0.2 ^c^	22.0 ± 0.6 ^a^
SGPAs *	23.8 ± 0.1 ^a^	18.7 ± 0.4 ^b^	23.5 ± 0.5 ^a^	24.3 ± 0.1 ^a^
SPAs *	634.0 ± 3.1 ^a^	301.4 ± 3.2 ^d^	390.2 ± 2.5 ^b^	362.9 ± 9.7 ^c^

Different letters in the same row indicate significant differences (*p* < 0.05).

**Table 3:** Analytical parameters of HPLC-ESI-MS/MS method.

Compound	Retention Time (min)	[M-H]^−^	Product Ions	Quantification Transition (*m*/*z*)	Fragmentor (V)	CE (V)
Gallic acid	1.53	169	125	169 → 125	108	12
Protocatechuic aldehyde	5.03	137	108	137 → 108	98	12
Dihydrofisetin glucoside	9.05	449	287, 259	449 → 287	131	16
Ellagic acid	12.25	301	284, 257	301→ 284	169	28
Ellagic acid hexoside 1	12.45	463	301, 169	463 → 301	169	28
Ellagic acid hexoside 2	12.82	463	301, 169	463 → 301	169	28
Kaempferol-glucoside	12.95	447	285	447 → 285	131	16
Quercetin-pentoside 1	14.24	433	301, 179, 151	433 → 151	131	16
Quercetin-pentoside 2	14.46	433	301, 179, 151	433 → 151	131	16
Quercetin	17.81	301	179, 151	301 → 151	131	16

**Table 4:** Concentrations (µg/g) of phenolic compounds in grape seeds enriched extracts from cv. Chardonnay (C) and Pignoletto (P) berries.

Compounds	CF1	CF2	PF1	PF2
	Phenolic Compounds (µg/g)			
Gallic acid	1788.7 ± 52.3 ^a^	1516.1 ±24.7 ^b^	832.4 ±10.8 ^c^	1490.6 ±19.9 ^b^
Protocatechuic aldehyde	108.8 ± 6.4 ^a^	62.0 ±2.9 ^b^	26.2 ±0.9 ^c^	58.0 ±1.5 ^b^
Dihydrofisetin glucoside	207.1 ± 2.2 ^a^	147.5 ±1.0 ^c^	152.2 ±1.2 ^b^	65.4 ±0.8 ^d^
Ellagic acid	1102.4 ± 6.9 ^b^	1401.7 ± 12.7 ^a^	< LOQ	< LOQ
Ellagic acid hexoside 1	22473.6 ± 24.3 ^b^	28726.7 ± 34.6 ^a^	12570.4 ± 21.8 ^d^	19509.3 ± 25.1 ^c^
Ellagic acid hexoside 2	3371.9 ± 8.9 ^c^	4719.2 ± 10.5 ^a^	2426.6 ± 6.1 ^d^	4407.6 ± 4.8 ^b^
Kaempferol-glucoside	55.5 ± 0.5 ^c^	77.5 ± 0.8 ^a^	35.2 ± 0.3 ^d^	66.3 ± 0.6 ^b^
Quercetin-pentoside 1	5.6 ± 0.2 ^b^	13.3 ± 0.5 ^a^	1.6 ± 0.2 ^c^	12.9 ± 0.3 ^a^
Quercetin-pentoside 2	4.0 ± 0.2 ^c^	11.8 ± 0.6 ^b^	2.0 ± 0.1 ^d^	20.7 ± 0.5 ^a^
Quercetin	85.0 ± 1.3 ^c^	177.5 ± 2.5 ^a^	10.3 ± 0.2 ^d^	98.8 ± 1.6 ^b^
Total	29202.5 ± 72.5 ^b^	36853.3 ± 69.8 ^a^	16056.8 ± 21.5 ^d^	25729.6 ± 20.7 ^c^

LOQ ellagic acid = 0.047 µg/mL; Different letters in the same row indicate significant differences (*p* < 0.05).
